# Cooling of the Nuclear Spin System of a Nanostructure by Oscillating Magnetic Fields

**DOI:** 10.3390/nano13142120

**Published:** 2023-07-20

**Authors:** Kirill V. Kavokin

**Affiliations:** Spin Optics Laboratory, St. Petersburg State University, Ulyanovskaya 1, St. Petersburg 198504, Russia; kkavokin@gmail.com

**Keywords:** spin, fluctuations, nanostructure, spin temperature, spin polarization

## Abstract

We propose a method of cooling nuclear spin systems of solid-state nanostructures by applying a time-dependent magnetic field synchronized with spin fluctuations. Optical spin noise spectroscopy is considered a method of fluctuation control. Depending on the mutual orientation of the oscillating magnetic field and the probe light beam, cooling might be either provided by dynamic spin polarization in an external static field or result from population transfer between spin levels without build-up of a net magnetic moment (“true cooling”).

## 1. Introduction

The energy transfer between nuclear spins and phonons in solids is known to be extremely slow, especially if the crystal lattice is kept at a cryogenic temperature, so that the spin-lattice relaxation time can reach hours [1,2]. At the same time, energy exchange between nuclear spins due to their magneto-dipole interaction occurs on the spin-spin relaxation timescale of approximately 0.1 millisecond. Off-diagonal elements of the density matrix of the nuclear spin system (NSS) decay at approximately the same time. As a result, the NSS reaches internal equilibrium, characterized by a spin temperature that can be many orders of magnitude lower than the lattice temperature, deep into the micro- or even nanoKelvin range [2]. Over the years that passed since the first experimental demonstration of the nuclear spin temperature [3], several methods were developed for cooling the NSS down to ultra-cryogenic temperatures. 

Application of an oscillating magnetic field to the nuclear spin system (NSS) is known to warm it up. If an external static magnetic field is applied, this effect amounts to the depolarization of nuclear spins and peaks up at NMR frequencies [1]. In a zero external field, it manifests itself as a decrease in the magnetic susceptibility of the NSS [4]. The question arises: Is it possible to create conditions under which an oscillating field would act in the opposite way, cooling the NSS? 

From general considerations, this might be possible if the oscillating field is synchronized with nuclear spin fluctuations. The rate of change of the NSS energy under the influence of the field $\vec{B}\left(t\right)$ equals:

(1) $$\frac{dE}{dt}=-\vec{M}\left(t\right)\cdot \frac{d\vec{B}\left(t\right)}{dt}$$

where $\vec{M}\left(t\right)$ is the total magnetic moment of the NSS. To provide a net change of the NSS energy, $\vec{M}\left(t\right)$ must be correlated with the field; in particular, if an oscillating magnetic field $B_{1}\left(t\right)=b_{1}\cos\left(\omega t\right)$ is applied, the averaged over the period $T=\frac{2\pi }{\omega }$ time derivative of the energy reads:

(2) $$\frac{dE}{dt}=-{\langle M_{B}\left(t\right)\cdot \frac{d}{dt}\left[b_{1}\cos\left(\omega t\right)\right]\rangle }_{T}=\frac{\omega b_{1}}{T}\int_{T}M_{B}\left(t\right)\sin\left(\omega t\right)$$

It is easy to show that in macroscopic solids, where spin fluctuations are negligible, the field-induced change of energy always results in heating up the NSS. Indeed, the mean magnetic moment induced by the field equals $\langle M_{B}\left(t\right)\rangle =b_{1}\left[\chi_{\omega }^{\prime }\cos\left(\omega t\right)+\chi_{\omega }^{\prime \prime }\sin\left(\omega t\right)\right]$, where $\chi_{\omega }^{\prime }$ and $\chi_{\omega }^{\prime \prime }$ are real and imaginary parts of the NSS susceptibility at the frequency ω. Now, as follows from Equation (2):

(3) $$\frac{dE}{dt}=\frac{1}{2}b_{1}^{2}\omega \chi^{\prime \prime }\left(\omega \right)=\frac{1}{4k_{B}\theta_{N}}b_{1}^{2}\omega^{2}\langle \delta M_{B}^{2}\rangle$$

where $\theta_{N}$ is the nuclear spin temperature. Here we used the well-known result of the fluctuation-dissipation theorem in the high-temperature limit [5]: $\chi^{\prime \prime }\left(\omega \right)=\frac{1}{2k_{B}\theta_{N}}\omega \langle \delta M_{B}^{2}\rangle$. One can see from Equation (3) that the oscillating field pumps energy into the NSS in case of positive $\theta_{N}$ and out of it in case of negative $\theta_{N}$. In both cases, the absolute value of $\theta_{N}$ increases, i.e., the interaction of the oscillating magnetic field with the average magnetic moment induced by this field always warms up the NSS.

However, if we are dealing with a finite-size NSS of a nanostructure, its magnetic moment includes a nonzero fluctuating part $\delta \vec{M}\left(t\right)$: $\vec{M}\left(t\right)=\langle \vec{M}\left(t\right)\rangle +\delta \vec{M}\left(t\right)$. Let us suppose that we can measure $\delta \vec{M}\left(t\right)$ in real time. This can be performed, for instance, by optical spin noise spectroscopy [6]. Moreover, we can apply an oscillating field in such a way that it would correlate with the nuclear spin fluctuation so that the average product of the time derivative of $\vec{B}\left(t\right)$ and the magnetic moment would be nonzero:

(4) $$\langle \delta \vec{M}\left(t\right)\cdot \frac{d\vec{B}}{dt}\rangle \neq 0$$

The resulted energy influx to the NSS would not depend on the NSS spin temperature, as distinct from the warm-up process, and would be linear in the magnetic field (and, consequently, its sign could be made positive or negative at the will of the experimentalist). This opens up the possibility of cooling the NSS to low positive or negative temperatures. 

In the following, two examples of experimental arrangements in which nuclear spins can be cooled by oscillating magnetic fields are considered. In the first example, the application of a constant magnetic field is necessary; here, cooling of the NSS is provided by the build-up of nuclear spin polarization parallel or antiparallel to this field. In the second example, the NSS cooling amounts to a population change in the energy levels of nuclear spins split by Zeeman, spin-spin, or quadrupole interactions and is not necessarily accompanied by net spin polarization (“true cooling”). 

## 2. Dynamic Spin Polarization by an Oscillating Magnetic Field in a Static External Field

**Figure 1:**
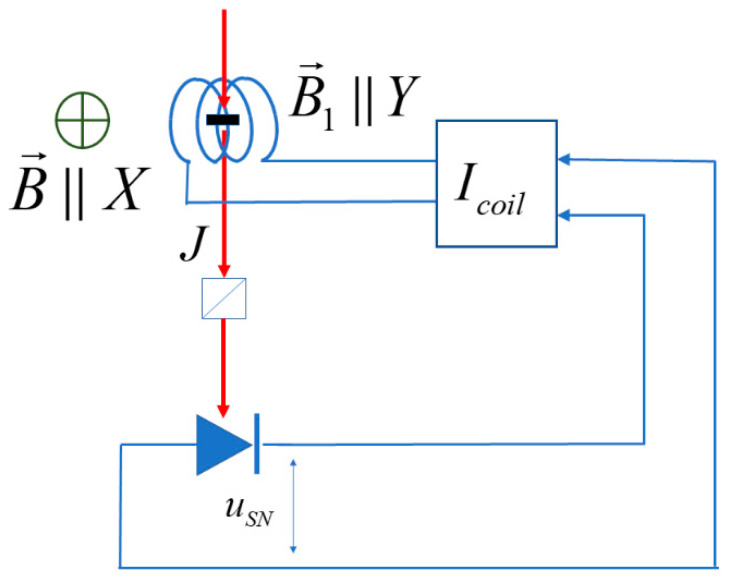
Scheme of the experiment on dynamic spin polarization in a constant magnetic field perpendicular to the structure axis. The red arrow shows the direction of the probe beam of linearly polarized light with fluence *J*. Fluctuations of its polarization plane induced by spin fluctuations in the sample, detected with the polarimetric device, form the spin noise signal $u_{sn}$ used to control the current in the magnetic coil that creates the time-dependent magnetic field $B_{1}\left(t\right)$.

We consider the experimental geometry shown in Figure 1.

A constant magnetic field $B_{X}=B$ is applied along the axis *X*. The *Z* component of the total magnetic moment of the probed volume, $M_{Z}$, is measured, and the time-dependent magnetic field is applied along *Y*.

(5) $$B_{1}\left(t\right)=\zeta M_{Z}\left(t\right)$$

Here, ζ is an adjustable transformation factor. One should note that Equation (1) is an idealization; in fact, the time-dependent field will inevitably contain an uncontrollable random contribution due, e.g., to the conversion of the photonic shot noise in the optical channel. The detrimental effect of this noise field will be considered later in Section 4.

**Figure 2:**
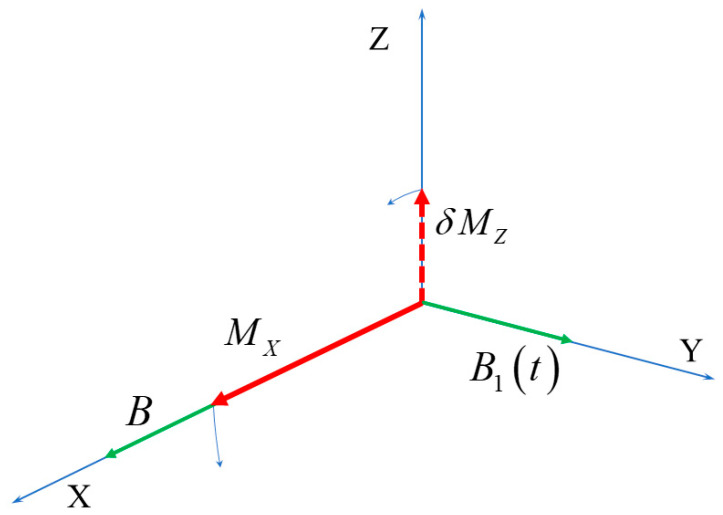
Schematic explanation of the dynamics of the nuclear magnetic moment under the magnetic field $B_{1}\left(t\right)$, correlated with the nuclear spin fluctuation. Green and red arrows show magnetic fields and magnetic moment components, correspondingly. The field $B_{1}\left(t\right)$ turns the *Z*-component of the fluctuating part of the nuclear magnetic moment, $\delta M_{Z}$, so that it feeds the regular magnetization along *X*. At the same time, $M_{X}$ turns in the XZ plane, so that $\delta M_{Z}$ decreases.

Qualitatively, the effect of the time-dependent field $B_{1}\left(t\right)$ on the nuclear magnetic moment is explained by the scheme shown in Figure 2. The vector of nuclear magnetic moment, $\vec{M}$, experiences Larmor precession about the total applied magnetic field $\vec{B}+{\vec{B}}_{1}\left(t\right)$. As $B_{1}\left(t\right)$ is correlated with $\delta M_{Y}$, the latter is always turned in the same direction, feeding the *X*-component of magnetization. At the same time, $M_{X}$ is turned so that it tends to compensate $\delta M_{Y}$, reducing the amplitude of the transverse spin fluctuation. According to the general theory of fluctuations [5], the latter is on average restored within the transverse relaxation time $T_{2}$. On the other hand, since the magnetic moment component along the constant field, $M_{X}$, decays with the longitudinal relaxation time $T_{1}$ that is much longer than $T_{2}$, $M_{X}$ accumulates and becomes much greater than the average fluctuation. 

The quantitative description of this process is provided by dynamic equations for the components of the magnetic moment $\vec{M}\left(t\right)$:

(6) $$\left\{\begin{array}{l} {\dot{M}}_{X}\left(t\right)=\gamma B_{1}\left(t\right)M_{Z}\left(t\right)-\frac{M_{X}\left(t\right)}{T_{1}}=\zeta \gamma M_{Z}^{2}\left(t\right)-\frac{M_{X}\left(t\right)}{T_{1}}\\ {\dot{M}}_{Y}\left(t\right)=-\gamma BM_{Z}\left(t\right)-\frac{M_{Y}\left(t\right)}{T_{2}}\\ {\dot{M}}_{Z}\left(t\right)=\gamma BM_{Y}\left(t\right)-\gamma B_{1}\left(t\right)M_{X}\left(t\right)-\frac{M_{Z}\left(t\right)}{T_{2}}=\gamma BM_{Y}\left(t\right)-\zeta \gamma M_{Z}\left(t\right)M_{X}\left(t\right)-\frac{M_{Z}\left(t\right)}{T_{2}} \end{array}\right.$$

where γ is the nuclear gyromagnetic ratio. 

In the following, we will develop these equations in the rotating-frame representation. It is the standard technique for the NMR theory, but as we are dealing with fluctuating magnetic moments, we chose to present a detailed derivation of the rotating-frame counterpart of Equation (6). The magnetic moment components in the laboratory frame are expressed via the magnetic moment components $M_{Y}^{\prime }\left(t\right)$ and $M_{Z}^{\prime }\left(t\right)$ in the coordinate frame rotating with the Larmor frequency, ω=γB, in the following way:

(7) $$\begin{array}{l} M_{Z}\left(t\right)=M_{Z}^{\prime }\left(t\right)\cos\omega t+M_{Y}^{\prime }\left(t\right)\sin\omega t\\ M_{Y}\left(t\right)=M_{Y}^{\prime }\left(t\right)\cos\omega t-M_{Z}^{\prime }\left(t\right)\sin\omega t \end{array}$$

Substituting these expressions into Equation (6), we obtain:

(8) $$\left\{\begin{array}{l} {\dot{M}}_{X}\left(t\right)=\zeta \gamma \left[M_{Z}^{\prime }{}^{2}\cos^{2}\omega t+2M_{Z}^{\prime }M_{Y}^{\prime }\cos\omega t\sin\omega t+M_{Y}^{\prime }{}^{2}\sin^{2}\omega t\right]-\frac{M_{X}\left(t\right)}{T_{1}}\\ {\dot{M}}_{Y}^{\prime }\left(t\right)\cos\omega t-{\dot{M}}_{Z}^{\prime }\left(t\right)\sin\omega t=-\frac{1}{T_{2}}\left[M_{Y}^{\prime }\left(t\right)\cos\omega t-M_{Z}^{\prime }\left(t\right)\sin\omega t\right]\\ {\dot{M}}_{Z}^{\prime }\left(t\right)\cos\omega t+{\dot{M}}_{Y}^{\prime }\left(t\right)\sin\omega t=-\left(\frac{1}{T_{2}}+\zeta \gamma M_{X}\left(t\right)\right)\left[M_{Y}^{\prime }\left(t\right)\cos\omega t-M_{Z}^{\prime }\left(t\right)\sin\omega t\right] \end{array}\right.$$

Multiplying the second equation in Equation (8) by cosωt and the third one by sinωt and adding up these two equations, we obtain the equation for the time derivative of ${\dot{M}}_{Y}^{\prime }\left(t\right)$:

(9) $${\dot{M}}_{Y}^{\prime }\left(t\right)=-\frac{1}{T_{2}}M_{Y}^{\prime }\left(t\right)-\zeta \gamma M_{X}\left(t\right)\left[M_{Z}^{\prime }\left(t\right)\cos\omega t\sin\omega t+M_{Y}^{\prime }\left(t\right)\sin^{2}\omega t\right]$$

Similarly, by multiplying the second equation in Equation (8) by sinωt and the third one by cosωt and subtracting, we obtain the equation for the time derivative of ${\dot{M}}_{Z}^{\prime }\left(t\right)$:

(10) $${\dot{M}}_{Z}^{\prime }\left(t\right)=-\frac{1}{T_{2}}M_{Z}^{\prime }\left(t\right)-\zeta \gamma M_{X}\left(t\right)\left[M_{Z}^{\prime }\left(t\right)\cos^{2}\omega t+M_{Y}^{\prime }\left(t\right)\sin\omega t\cos\omega t\right]$$

The first equation in Equations (8) together with Equations (9) and (10) forms the system of equations for the magnetic moment components in the rotating frame:

(11) $$\left\{\begin{array}{l} {\dot{M}}_{X}\left(t\right)=\zeta \gamma \left[M_{Z}^{\prime }{}^{2}\cos^{2}\omega t+2M_{Z}^{\prime }M_{Y}^{\prime }\cos\omega t\sin\omega t+M_{Y}^{\prime }{}^{2}\sin^{2}\omega t\right]-\frac{M_{X}\left(t\right)}{T_{1}}\\ {\dot{M}}_{Y}^{\prime }\left(t\right)=-\frac{1}{T_{2}}M_{Y}^{\prime }\left(t\right)-\zeta \gamma M_{X}\left(t\right)\left[M_{Z}^{\prime }\left(t\right)\cos\omega t\sin\omega t+M_{Y}^{\prime }\left(t\right)\sin^{2}\omega t\right]\\ {\dot{M}}_{Z}^{\prime }\left(t\right)=-\frac{1}{T_{2}}M_{Z}^{\prime }\left(t\right)-\zeta \gamma M_{X}\left(t\right)\left[M_{Z}^{\prime }\left(t\right)\cos^{2}\omega t+M_{Y}^{\prime }\left(t\right)\sin\omega t\cos\omega t\right] \end{array}\right.$$

By using the identities $\cos^{2}\omega t=\frac{1}{2}\left(1+\cos2\omega t\right)$ and $\sin\omega t\cos\omega t=\frac{1}{2}\sin2\omega t$, and neglecting terms oscillating at double frequency, Equation (11) is reduced to:

(12) $$\left\{\begin{array}{l} {\dot{M}}_{X}\left(t\right)=\frac{1}{2}\zeta \gamma \left[M_{Z}^{\prime }{}^{2}+M_{Y}^{\prime }{}^{2}\right]-\frac{M_{X}\left(t\right)}{T_{1}}\\ {\dot{M}}_{Y}^{\prime }\left(t\right)=-\left[\frac{1}{T_{2}}+\frac{1}{2}\zeta \gamma M_{X}\left(t\right)\right]M_{Y}^{\prime }\left(t\right)\\ {\dot{M}}_{Z}^{\prime }\left(t\right)=-\left[\frac{1}{T_{2}}+\frac{1}{2}\zeta \gamma M_{X}\left(t\right)\right]M_{Z}^{\prime }\left(t\right) \end{array}\right.$$

Averaging the first equation in Equation (12) yields the equation for the mean value of $M_{X}\left(t\right)$:

(13) $$\frac{d}{dt}\langle M_{X}\left(t\right)\rangle =\frac{1}{2}\zeta \gamma \left[\langle \delta M_{Z}^{\prime }{}^{2}\left(t\right)\rangle +\langle \delta M_{Y}^{\prime }{}^{2}\left(t\right)\rangle \right]-\frac{\langle M_{X}\left(t\right)\rangle }{T_{1}}$$

where $\delta M_{Z}^{\prime }$ and $\delta M_{Y}^{\prime }$ are fluctuations of the *Z* and *Y* components of the magnetic moment in the rotating frame, whose mean values remain zero. Further, assuming $\frac{1}{2}\zeta \gamma \delta M_{X}\left(t\right)T_{2}<<1$, where $\delta M_{X}\left(t\right)$ is the fluctuation of the *X*-component of the magnetic moment, one can replace $M_{X}\left(t\right)$ in the second and third equations in Equation (12) with their average given by Equation (13).

The equations for fluctuations $\delta M_{Z}^{\prime }$ and $\delta M_{Y}^{\prime }$ are obtained from the second and third equations in Equation (12) by adding to their right-hand sides Langevin forces $\xi_{Z}\left(t\right)$ and $\xi_{Y}\left(t\right)$ [5] with correlation functions:

(14) $$\begin{array}{l} \langle \xi_{Z}\left(t\right)\xi_{Z}\left(t^{\prime }\right)\rangle =a_{Z}\delta \left(t-t^{\prime }\right)\\ \langle \xi_{Y}\left(t\right)\xi_{Y}\left(t^{\prime }\right)\rangle =a_{Y}\delta \left(t-t^{\prime }\right)\\ \langle \xi_{Z}\left(t\right)\xi_{Y}\left(t^{\prime }\right)\rangle =0 \end{array}$$

The factors $a_{Z}$ and $a_{Y}$ are found from the condition that in the absence of the time-dependent field, i.e., when ζ=0, the mean squared values $\delta M_{Z}^{\prime }$ and $\delta M_{Y}^{\prime }$ take their thermodynamically equilibrium form. In the case of weak spin polarization, i.e., when $\langle M_{Z}\rangle <<N\hbar \gamma I$, where *I* is the spin of a single nucleus and *N* is the number of nuclei in the probed volume.

(15) $$\langle \delta M_{Z}^{\prime }{}^{2}\rangle =\langle \delta M_{Y}^{\prime }{}^{2}\rangle =N\frac{I\left(I+1\right)}{3}{\left(\hbar \gamma \right)}^{2}$$

The correlation function of a random value *x*(*t*) described by the Langevin equation $\dot{x}\left(t\right)=-\lambda x\left(t\right)+\xi_{x}\left(t\right)$ equals $\langle x\left(0\right)x\left(\tau \right)\rangle =\frac{a_{x}}{2\lambda }\exp\left(-\lambda \tau \right)$ [5]. From Equations (12), (14), and (15), we then find:

(16) $$a_{Y}=a_{Z}=2N{\left(\hbar \gamma \right)}^{2}\frac{I\left(I+1\right)}{3}\cdot \frac{1}{T_{2}}$$

At nonzero ζ, $\lambda =\frac{1}{T_{2}}+\frac{1}{2}\zeta \gamma \langle M_{X}\rangle$. Therefore,

(17) $$\begin{array}{l} \langle M_{Z}^{\prime }\left(0\right)M_{Z}^{\prime }\left(\tau \right)\rangle =\langle M_{Y}^{\prime }\left(0\right)M_{Y}^{\prime }\left(\tau \right)\rangle =\\ =N{\left(\hbar \gamma \right)}^{2}\frac{I\left(I+1\right)}{3}{\left(1+\frac{1}{2}\zeta \gamma \langle M_{X}\rangle T_{2}\right)}^{-1}\exp\left[-\tau \left(\frac{1}{T_{2}}+\frac{1}{2}\zeta \gamma \langle M_{X}\rangle \right)\right] \end{array}$$

The equation for $\langle M_{X}\rangle$ (see Equation (13)) now takes the form:

(18) $$\frac{d}{dt}\langle M_{X}\left(t\right)\rangle =N{\left(\hbar \gamma \right)}^{2}\frac{I\left(I+1\right)}{3}\cdot \zeta \gamma {\left(1+\frac{1}{2}\zeta \gamma \langle M_{X}\rangle T_{2}\right)}^{-1}-\frac{\langle M_{X}\left(t\right)\rangle }{T_{1}}$$

Its stationary solution is:

(19) $$\langle M_{X}\rangle =\frac{-1+\sqrt{1+2{\left(\zeta \gamma \right)}^{2}T_{1}T_{2}NI\left(I+1\right){\left(\hbar \gamma \right)}^{2}/3}}{\zeta \gamma T_{2}}$$

The spin polarization of nuclei in the probed volume is then equal to:

(20) $$p=\frac{\langle M_{X}\rangle }{\hbar \gamma IN}=p_{0}\frac{-1+\sqrt{1+2{\left(\zeta /\zeta_{0}\right)}^{2}}}{\zeta /\zeta_{0}}$$

where

(21) $$p_{0}=\sqrt{\frac{I+1}{3IN}\cdot \frac{T_{1}}{T_{2}}}$$

and

(22) $$\zeta_{0}=\frac{1}{\hbar \gamma^{2}\sqrt{NI\left(I+1\right)T_{1}T_{2}/3}}$$

At small ζ:

(23) $$p\approx p_{0}\zeta /\zeta_{0}$$

At large ζ the nuclear polarization saturates, approaching the value $p_{0}\sqrt{2}$, which is $\sqrt{2\frac{T_{1}}{T_{2}}}$ times larger than its mean squared fluctuation at thermodynamic equilibrium.

**Figure 3:**
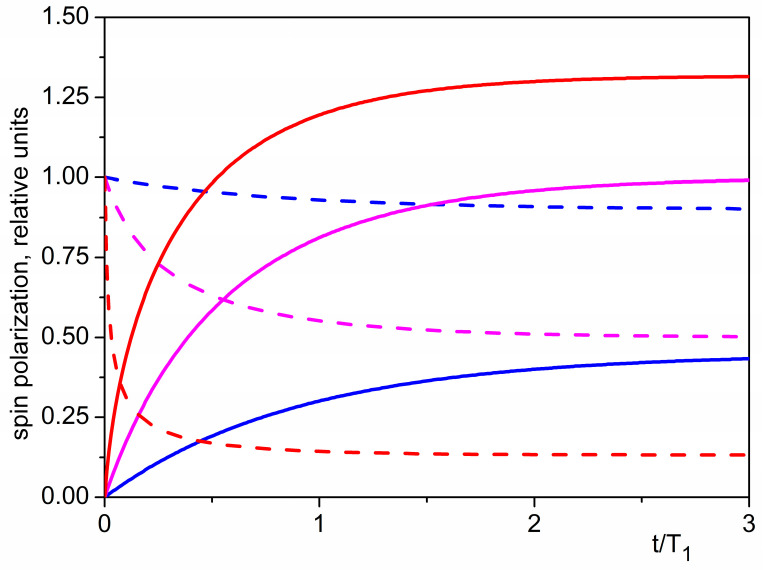
Evolution of the mean spin polarization along the static field $p/p_{0}$ (solid curves) and of the mean squared transverse fluctuation in relation to its equilibrium value (dashed curves), after switching on the magnetic field $B_{1}\left(t\right)$, for different values of the transformation coefficient ζ. Blue curves: $\zeta =0.5\zeta_{0}$; magenta curves: $\zeta =2\zeta_{0}$; red curves: $\zeta =10\zeta_{0}$.

Figure 3 shows the time evolution of mean nuclear polarization after switching on the time-dependent magnetic field $B_{1}\left(t\right)$, obtained from the numerical solution of Equation (18), as well as the time dependence of mean squared transverse spin fluctuations, given by Equation (17) at τ=0.

One can easily check that Equation (18) indeed describes the cooling process of the nuclear spin system. Multiplying it by the constant field B||X, we arrive at the equation of the energy balance in the NSS:

(24) $$\frac{dE}{dt}=q-\frac{E}{T_{1}}$$

where q is the energy influx into the NSS. In the limit of small ζ, when transverse spin fluctuations are not suppressed,

(25) $$q=-\zeta N{\left(\hbar \gamma \right)}^{2}\frac{I\left(I+1\right)}{3}\cdot \gamma B=-\zeta \omega \cdot N{\left(\hbar \gamma \right)}^{2}\frac{I\left(I+1\right)}{3}$$

As follows from Equation (7),

(26) $$\langle \delta M_{Y}\frac{dB_{1}}{dt}\rangle =\zeta \langle \delta M_{Y}\frac{d}{dt}\delta M_{Z}\rangle =\zeta \omega \cdot \frac{1}{2}\left(\langle \delta {M_{X}^{\prime }}^{2}\rangle +\langle \delta {M_{Y}^{\prime }}^{2}\rangle \right)=\zeta \omega N{\left(\hbar \gamma \right)}^{2}\frac{I\left(I+1\right)}{3}$$

By comparing Equations (25) and (26), we find that

(27) $$q=-\langle \delta M_{Y}\frac{dB_{1}}{dt}\rangle$$

is in full agreement with Equation (1). However, we note that cooling in this experimental geometry occurs via dynamic polarization: transverse spin fluctuations are turned so as to build up a net magnetization along X, and the polarity of this magnetization is defined by the sign of the transformation coefficient ζ and does not depend on the polarity of the static field *B*. This is similar to what happens when nuclear spins are cooled via dynamic polarization by electrons [7]: the spin temperature is reduced because the Zeeman energy of the NSS changes as spins are polarized along or opposite to the static external field. One can change the sign of the Zeeman energy acquired by the NSS and, therefore, the sign of spin temperature by changing the polarity of the static field. No cooling is possible if there is no static field, because in that case the Zeeman energy would be zero.

## 3. “True Cooling” of Nuclear Spins by Oscillating Magnetic Fields

**Figure 4:**
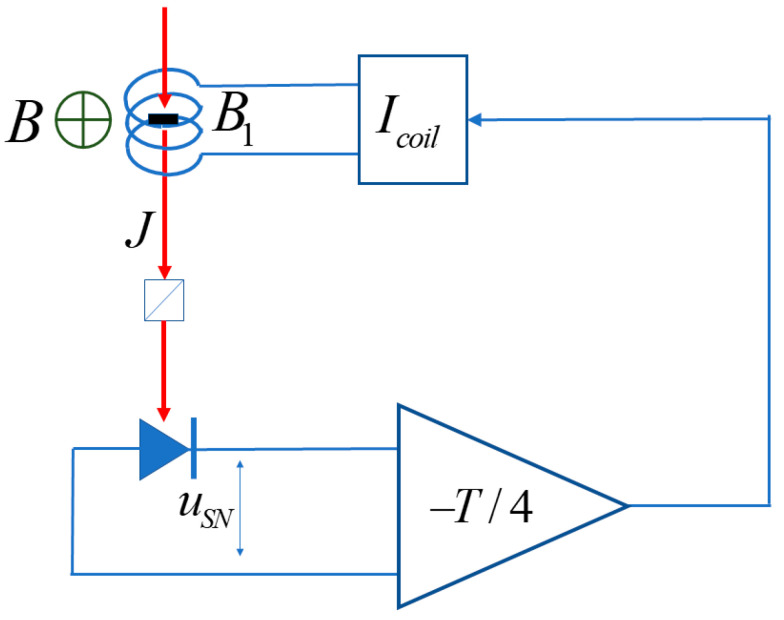
Experimental arrangement for “true” nuclear spin cooling in an external field. The red arrow shows the direction of the probe beam of linearly polarized light with fluence *J*. The time-dependent field $B_{1}\left(t\right)$ is applied parallel to the probe beam, with the $-T/4=-\pi /\left(2\left|\gamma B\right|\right)$ phase shift between the field and the optical spin noise signal being provided by the electronics.

In this section, we consider the experimental arrangement that allows one to cool nuclear spins to a certain sign of spin temperature irrespective of the polarity of the external static field. As distinct from the case considered in the previous Section, the field $B_{1}\left(t\right)$ is applied parallel to the probe beam along Z (see Figure 4). An electronic circuit ensures that $B_{1}\left(t\right)$ is delayed with respect to the magnetization fluctuation by a quarter period of spin precession in the static field *B* directed along X.

The dynamics of the cartesian components of the magnetic moment in this case are described by the following equations:

(28) $$\left\{\begin{array}{l} {\dot{M}}_{X}\left(t\right)=\gamma B_{1}\left(t\right)M_{Y}\left(t\right)-\frac{M_{X}\left(t\right)}{T_{1}}\\ {\dot{M}}_{Y}\left(t\right)=-\gamma BM_{Z}\left(t\right)+\gamma B_{1}\left(t\right)M_{X}\left(t\right)-\frac{M_{Y}\left(t\right)}{T_{2}}\\ {\dot{M}}_{Z}\left(t\right)=\gamma BM_{Y}\left(t\right)-\frac{M_{Z}\left(t\right)}{T_{2}} \end{array}\right.$$

Presenting the transverse components in the form given by Equation (7), we find that:

(29) $$\begin{array}{l} B_{1}\left(t\right)=\zeta M_{Z}\left(t-T/4\right)=\zeta \left\{M_{Z}^{\prime }\left(t\right)\cos\left[\omega \left(t-T/4\right)\right]+M_{Y}^{\prime }\left(t\right)\sin\left[\omega \left(t-T/4\right)\right]\right\}=\\ =\zeta \left\{M_{Z}^{\prime }\left(t\right)\cos\left[\gamma Bt-\frac{\pi B}{2\left|B\right|}\right]+M_{Y}^{\prime }\left(t\right)\sin\left[\gamma Bt-\frac{\pi B}{2\left|B\right|}\right]\right\}=\\ =\zeta \frac{B}{\left|B\right|}\left[M_{Z}^{\prime }\left(t\right)\sin\left(\omega t\right)-M_{Y}^{\prime }\left(t\right)\cos\left(\omega t\right)\right]=-\zeta \frac{B}{\left|B\right|}M_{Y}\left(t\right) \end{array}$$

Substituting this result into the first equation in Equation (29) and taking the ensemble average, one obtains the equation for the *X*-component of the magnetic moment:

(30) $$\langle {\dot{M}}_{X}\left(t\right)\rangle =-\zeta \gamma \frac{B}{\left|B\right|}\cdot \frac{1}{2}\left[\langle {M_{Y}^{\prime }}^{2}\left(t\right)\rangle +\langle {M_{Z}^{\prime }}^{2}\left(t\right)\rangle \right]-\frac{\langle M_{X}\left(t\right)\rangle }{T_{1}}$$

It is easy to show that the equations for mean squared transverse components, derived from Equation (29), appear to be the same as in the previous Section. Therefore, the absolute value of the spin polarization will be given by Equation (20). However, comparing Equations (13) and (30), one can see that the sign of $\langle M_{X}\left(t\right)\rangle$, which builds up under the influence of the field $B_{1}\left(t\right)$, now depends on the polarity of *B*. Consequently, the sign of Zeeman energy does not depend on the polarity of *B* and is solely determined by the sign of the transformation coefficient ζ.

Imagine now that each nuclear spin is subjected to a local magnetic field with the strength $\left|B\right|$, besides polarities of these fields are random. It follows from Equation (30) that the average magnetization of the NSS in this case will remain close to zero, while the energy will increase in absolute value, and consequently the absolute value of spin temperature will decrease. This is what we would like to call “true cooling”: the spin temperature is reduced in absolute value while no net magnetization builds up.

In real nanostructured solids, a similar situation can occur due to spin-spin or quadrupole interactions. If no external magnetic field is applied, the energy levels of the nuclear spin can still be split by internal magnetic fields created by other nuclear spins or, in the case of spins *I* > 1/2, by quadrupole interaction with electric field gradients. Such gradients are ubiquitous in nanostructures due to almost unavoidable internal strains. In particular, quadrupole splitting results in the appearance of distinct peaks at frequencies of the order of 10 kHz, as clearly observed in the nuclear spin warm-up spectra [8] in GaAs. The splitting can become greater in intentionally strained structures, e.g., self-assembled quantum dots [9,10,11]. If this splitting is much larger than the characteristic energy of dipole-dipole interactions that defines the transverse relaxation time $T_{2}$, one can describe the dynamic of populations at these two levels by a 2 × 2 density matrix, which is conveniently expanded over the Pauli matrices. The coefficients of this expansion can be considered components of the pseudospin ½ [12]. This way, the theoretical description of spin dynamics of the pair of quadrupole-split levels reduces to solving a system of equations analogous to Equation (28), where spin components along Z, X, and Y are replaced with the population difference of the two levels, real and imaginary parts of the off-diagonal element of the density matrix, correspondingly. Therefore, the overall picture of cooling quadrupole-split nuclear spins should be similar to that of cooling in an external static field, with the cooling rate being dependent on specific matrix elements of the field $B_{1}\left(t\right)$ between quadrupole-split levels.

As shown in Ref. [13], quadrupole, dipole-dipole, and Zeeman reservoirs in semiconductor structures are effectively coupled even at quadrupole splitting exceeding 10 kHz. Therefore, the “true” cooling of the quadrupole reservoir would result in establishing a low spin temperature in the entire NSS, which can be detected by measuring its susceptibility to weak probe magnetic fields via, e.g., Faraday rotation induced by the Overhauser field [14].

## 4. Limitations of the Method and Numerical Estimates

**Figure 5:**
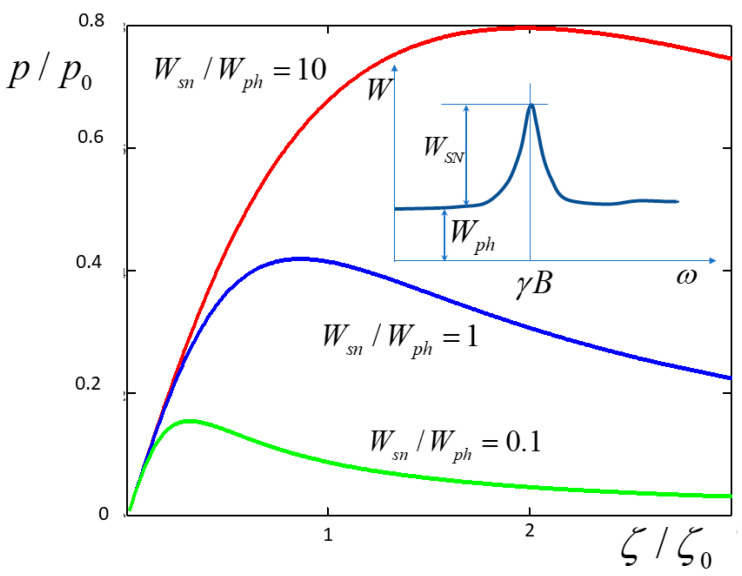
Spin polarization vs. the transformation coefficient ζ for different ratios of spectral power densities of spin noise $W_{sn}$ (at the peak) and of background photonic noise $W_{ph}$. Inset: typical spectrum of spin noise in a transverse magnetic field over the background of photonic noise.

The main limitation of the method comes from the background noise in the optical channel, which, being amplified and converted into the current in the magnetic coil, gives rise to a noise magnetic field that warms up the nuclear spin system. Up-to-date spin noise spectroscopy can successfully fight all sources of noise except the shot noise of photons in the probe beam [6]. This photonic noise results in fluctuations of the Faraday rotation angle, with the flat spectral power density inversely proportional to the fluence of the probe beam. A typical spectrum of Faraday rotation noise of a spin system in a transverse magnetic field is shown in the inset to Figure 5.

If the spectral power density (SPD) of photonic noise is $W_{ph}$ and that of the spin noise at the resonance peak is $W_{sn}$, the transformation of the photonic noise by the circuitry results in a random magnetic field with the SPD equal to:

(31) $$B_{ph}^{2}\left(\omega \right)\approx \frac{W_{ph}}{W_{sn}}\zeta^{2}\hbar^{2}\gamma^{2}\frac{NI\left(I+1\right)}{3}\cdot T_{2}$$

This random field induces depolarization of nuclear spins at the rate of:

(32) $$\frac{1}{T_{ph}}=\gamma^{2}B_{ph}^{2}\left(\omega \right)\approx \frac{W_{ph}}{W_{sn}}\zeta^{2}\hbar^{2}\gamma^{4}\frac{NI\left(I+1\right)}{3}\cdot T_{2}=\frac{W_{ph}}{W_{sn}}\frac{\zeta^{2}}{\zeta_{0}^{2}}\cdot \frac{1}{T_{1}}$$

where $\zeta_{0}^{2}$ is given by Equation (22). Therefore, to take into account spin depolarization, or warm-up, due to the photonic noise, one should replace $T_{1}$ in Equations (19)–(21) with $T_{1}^{*}$ defined as:

(33) $$T_{1}^{*}=T_{1}\cdot {\left(1+\frac{W_{ph}}{W_{sn}}\frac{\zeta^{2}}{\zeta_{0}^{2}}\right)}^{-1}$$

The dependences of spin polarization on the transformation coefficient ζ for different ratios of spectral power densities of the spin noise and the background photonic noise are plotted in Figure 5. One can see that the warm-up due to the background noise results in a decrease in polarization at large ζ. The polarization that can be reached at optimal $\zeta ∼\zeta_{0}$ rather weakly depends on $W_{sn}/W_{ph}$; in fact, it amounts to a considerable fraction of $p_{0}$ once the spin noise peak is discernible over the photonic noise background.

To estimate the effect in numbers, one needs to consider a specific object. The possibility of detecting the nuclear spin fluctuations optically has already been demonstrated experimentally in bulk GaAs [15]. We propose to use GaAs/AlGaAs microcavity structures, which vastly improve the sensitivity of the method [16,17]. In order to estimate the efficiency of nuclear spin cooling by oscillating fields, we assume the use of an optical microcavity with a GaAs active layer, similar to the one studied in Ref. [14]. With the thickness of the active layer of 0.35 μm and the beam diameter of 2 μm, the probed volume is approximately 1 ${\mu m}^{3}$ and the number of nuclei in the probed volume is $N\approx 4\times 10^{10}$. The probe beam makes about 1000 round trips inside the cavity, which results in an effective optical path $L_{eff}\approx 0.7$ mm. The Faraday rotation angle $\theta_{f}$, induced by the Overhauser field of nuclear fluctuations, $B_{Nf}$, equals:

(34) $$\theta_{f}=V_{N}L_{eff}B_{Nf}$$

where the nuclear Verdet constant is $V_{N}\approx 0.1$ mrad/(cm·G) [14]. The mean squared Overhauser field of the projection of the nuclear spin fluctuation on the structure axis Z equals:

(35) $$\langle B_{NfZ}^{2}\rangle =N\frac{I\left(I+1\right)}{3}{\left(\frac{b_{N}}{IN}\right)}^{2}=\frac{I+1}{3IN}b_{N}^{2}$$

where $b_{N}\approx$ 5.3 T is the maximum Overhauser field reached when all the nuclear spins in GaAs are fully polarized.

Thus, the mean squared fluctuation of the Faraday angle equals:

(36) $$\langle \theta_{f}^{2}\rangle \approx {\left(V_{N}L_{eff}\right)}^{2}\frac{I+1}{3IN}b_{N}^{2}$$

Substituting here the structure parameters, we obtain $\langle \theta_{f}^{2}\rangle \approx 3\times 10^{-12}{\;\mathrm{rad}}^{2}$. The frequency range of the fluctuating Faraday signal induced by nuclear spins is determined by the inverse of the spin-spin relaxation time $T_{2}\approx 10^{-4}s$. The mean squared fluctuation of the polarization plane due to the photonic noise of the probe beam with intensity *J* in the frequency band 1/*T*_2_ is:

(37) $$\langle \theta_{ph}^{2}\rangle \approx \frac{1}{JT_{2}}$$

Taking these two values equal, we obtain the light intensity under which the spin noise has the same SPD as the photonic one, $J\approx 3\times 10^{15}\;\mathrm{ph}/s$, which corresponds, with the photon energy of 1.4 eV, to the probe beam transmitted power of 0.7 mW. This is a realistic value for this kind of experiment.

With the typical $T_{1}=100$ s, one gets, according to Equation (21), $p_{0}\approx 0.004$ that corresponds, for $W_{sn}/W_{ph}=1$, to the maximum polarization $p\approx 1.6\times 10^{-3}$ and maximum Overhauser field of 80 G. Such effective fields are easily detected and measured with optical methods, e.g., by Faraday rotation [14]. These values of polarization and Overhauser field are reached at $\zeta \approx \zeta_{0}$, which corresponds to the amplitude of the field $B_{1}\left(t\right)$ approximately equal to $\sqrt{\frac{NI\left(I+1\right)}{3}}\hbar \gamma \zeta_{0}=\frac{1}{\gamma \sqrt{T_{1}T_{2}}}\approx 1$ mG.

On the whole, the estimated values of experimental parameters and the expected magnitude of the outcome suggest that observation of the effect in GaAs-based microcavity structures is quite realistic. Using more sophisticated structures, e.g., ones with quantum dots in the microcavity, might further enhance the achievable nuclear spin polarization via reducing the number of spins in the probed volume; however, evaluation of the magnitude of spin polarization in such structures can be difficult because of, e.g., inhomogeneous strains and interfacial effects.

## 5. Conclusions

We have proposed a theoretical background for the development of a new method of nuclear spin cooling that does not involve dynamic polarization by electrons. In fact, the NSS is cooled by an “optical Maxwell demon”, which monitors nuclear spin fluctuations and controls the external magnetic field in a way to pump energy into or out of the NSS. In one of the examples considered, a net nuclear magnetization is built up along a certain direction defined by the experimental geometry, similar to the dynamic polarization of spin-polarized electrons. In the other experimental arrangement, cooling that is not accompanied by magnetization build-up, or “true cooling”, can be realized. Numerical estimates for a GaAs-based microcavity structure demonstrate the feasibility of the proposed method. The efficiency of spin cooling can be enhanced by using a quantum dot structure with a reduced total number of nuclear spins.

## Data Availability

Data sharing not applicable.
